# Targeted cytokine delivery: cell therapy to remodel the pre-metastatic niche

**DOI:** 10.1038/s41392-021-00694-1

**Published:** 2021-07-25

**Authors:** Michael A. Morgan, Lucas Lange, Axel Schambach

**Affiliations:** 1 Institute of Experimental Hematology, Hannover Medical School, Hannover, Germany; 2 REBIRTH Research Center for Translational Regenerative Medicine, Hannover Medical School, Hannover, Germany; 3 Division of Hematology/Oncology, Boston Children’s Hospital, Harvard Medical School, Boston, MA USA

**Keywords:** Cancer microenvironment, Cancer therapy, Molecular medicine, Cell biology

Tumor metastases present an often difficult to treat therapeutic challenge for cancer patients and their caregivers. The recent paper by the Kaplan group (Kaczanowska et al.) exploited the natural capacity of monocytes or a subset of monocytes to migrate to tumor sites and pre-metastatic niches to deliver IL-12 in a novel anticancer approach designed to stimulate local immune responses.^1^ This raises the possibility to simultaneously fight cancer on at least two critical fronts, (1) elimination of the primary tumor and (2) inhibition of newly developing pre-metastatic lesions.

Kaczanowska et al. used a syngeneic orthotopic tumor model of rhabdomyosarcoma that is known to metastasize to the lung. Rhabdomyosarcoma is an aggressive muscle-derived soft-tissue cancer and less than one-fifth of patients with metastatic rhabdomyosarcoma can be cured. Transcriptomic and flow cytometric analyses to compare pre-metastatic lungs with naive lungs were consistent with an immunosuppressive microenvironment mediated by myeloid cells and a lack of lymphocytes in pre-metastatic lungs. In an effort to generate a cell therapy to remodel the pre-metastatic tumor niche, Kaczanowska et al. transduced murine bone marrow cells enriched for hematopoietic progenitor cells with a self-inactivating (SIN) lentiviral vector that expressed IL-12 via the EF1α promoter to generate genetically engineered murine myeloid cells that they call IL12-GEMys.

Intravenous administration of the heterogeneous IL12-GEMy cell product, which had scRNA-seq patterns consistent with macrophages and monocytes, showed peak IL-12 and IFN-γ levels in the lungs 4 days and 7 days after cell application, respectively. Treatment with IL12-GEMys resulted in significantly reduced metastasis, reduced primary tumors, and improved survival compared to control-treated mice. Application of IL12-GEMys prior to tumor resection also led to prolonged survival compared to mice that only had surgery. Furthermore, infusion of 1 × 10^6^ IL12-GEMys had only transient anticancer effects in a few mice, while treatment with 8 × 10^6^ IL12-GEMys led to longer anti-tumor activity in all tested mice. Interestingly, a combination of IL12-GEMys with chemotherapy was especially efficacious in pre-clinical murine tumor models, even resulting in cure in some mice. As it is always of interest to explore how broadly applicable a particular therapeutic strategy may be, Kaczanowska et al. also showed that their IL12-GEMys could inhibit liver metastasis from an aggressive pancreatic cancer model.

As a cellular mechanism, the authors found that IL12-GEMys activate T-cell responses. Depletion of CD8^+^ cells negated beneficial effects of IL12-GEMy treatment, while depletion of CD4^+^ cells led to only partially reduced anti-tumor activity. In a further step toward clinical translation, the authors showed that cyclophosphamide and fludarabine preconditioning resulted in increased amounts of IL12-GEMys in the lungs with strong expansion of PD-1^+^Lag3^-^CD8^+^ T cells in the lung and tumor. The finding that cured mice that were re-challenged with the original tumor line did not develop tumors is evidence that IL12-GEMy treatment led to the generation of functional anti-tumor memory T cells.

The idea to combine gene therapy principles to engineer cells to express immunologically active proteins like IL-12 with the ability of myeloid cells to home to pre-metastatic sites or even infiltrate immunologically cold tumors is a great extension to the rapidly developing field of immune therapy. Many recent breakthroughs in immune therapeutic strategies were reported in the last two decades, such as adoptive T-cell therapies, including chimeric antigen receptor (CAR) T-cell-based approaches. CAR T cells are T cells that have been genetically modified to express receptors that recognize a specific neoantigen or tumor-associated antigen and that activate the cytotoxic activity of the T cell upon antigen binding. While this strategy has shown clinical efficacy in the treatment of lymphoid-derived cancers, solid tumors have presented additional challenges.

For example, loss of immune cell activity is thought to be due to the immunosuppressive tumor microenvironment (TME), including pro-tumor cell populations such as M2 macrophages, myeloid-derived suppressor cells, and regulatory T cells. Therefore, many efforts have been invested in the development of strategies to increase anti-tumor activity of immune cells. As described by Kaczanowska et al., directed delivery of cytokines can help to remodel the TME and stimulate anti-tumor immune cell activity without detectable adverse effects. Whereas Kaczanowska et al. used GEMys to deliver IL-12 to pre-metastatic sites, other cytokine delivery methods have also shown promise (Fig. 1). Intratumoral application of IL-12 was shown to enhance the activity of CAR T cells designed to target the tumor-associated antigen epidermal growth factor receptor variant III in an immunocompetent, orthotopic glioblastoma multiforme mouse model.^2^ In addition to improved CAR T cell activity, local IL-12 application led to lower Treg cell numbers, higher proinflammatory CD4^+^ T cells, and greater activation of myeloid cells in the remodeled TME, with only limited systemic effects. Another approach used “T cells redirected for universal cytokine-mediated killing”, or TRUCKs, in which signaling from a CAR T cell upon binding to the target antigen leads to activation of an inducible gene expression cassette to deliver the cytokine of choice (e.g., IL-12, IL-18….) to the tumor microenvironment.^3–5^ This strategy was shown to recruit macrophages and other immune cells to the tumor site and to increase anti-tumor effects. However, it is important to point out that uncontrolled or leaky IL-12 expression could result in severe and potentially fatal toxicities, such as high fevers, liver dysfunction, and hemodynamic instability.

**Fig. 1:**
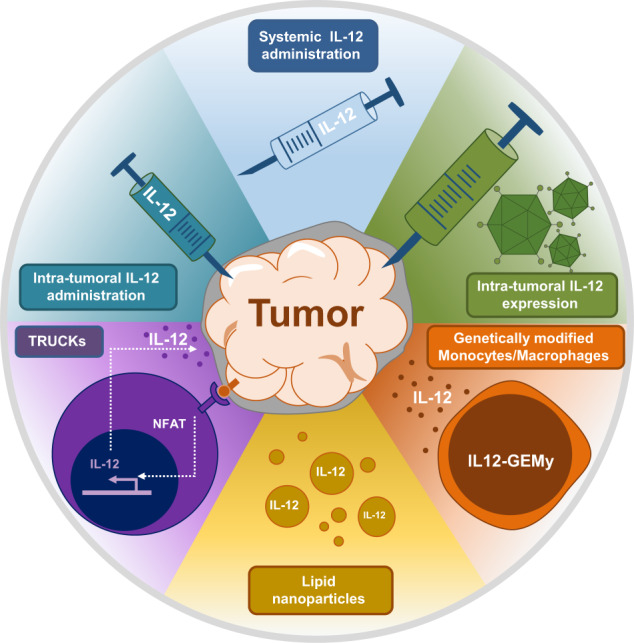
Strategies to deliver cytokines to improve anticancer therapies. Cytokines (e.g., IL-12) that can recruit immune cells to the tumor or pre-metastatic sites continue to be tested in therapeutic oncology settings. Several methods to apply IL-12 are shown here, including the use of lipid nanoparticles, “T cells redirected for universal cytokine-mediated killing” (TRUCKs), intratumoral administration, systemic application, intratumoral expression, and genetically engineered myeloid cells (IL12-GEMys)

Kaczanowska et al. also presented work toward translation of this concept from the mouse tumor model to human cells. Here, human IL12-GEMys were generated by lentiviral vector transduction of the SC cell line that was derived from human peripheral blood mononuclear cells. Co-culture of human IL12-GEMys with human lymphocytes led to significantly increased secretion of IFN-γ, indicating activation of human lymphocytes. It will be of interest to follow the next steps of this work and see how effective the IL12-GEMys are when applied to solid tumor patients as the cancer cell populations in human tumors are often more heterogeneous than those in murine models. Will the cell line be the most suitable source of human IL12-GEMys for clinical application or will future work rather turn to hematopoietic stem and progenitor cells as Kaczanowska et al. did in the murine setting? Perhaps a renewable allogeneic cell source for off-the-shelf precision medicine cell therapeutics will lead to a more homogenous cell product with equal or improved anticancer activity. Regardless, the work by Kaczanowska et al. represents an important step in the right direction for the development of cell therapeutics to stop cancer metastases.

## References

[1] Kaczanowska S (2021). Genetically engineered myeloid cells rebalance the core immune suppression program in metastasis. Cell.

[2] Agliardi G (2021). Intratumoral IL-12 delivery empowers CAR-T cell immunotherapy in a pre-clinical model of glioblastoma. Nat. Commun..

[3] Chmielewski M, Abken H (2012). CAR T cells transform to trucks: chimeric antigen receptor-redirected T cells engineered to deliver inducible IL-12 modulate the tumour stroma to combat cancer. Cancer Immunol. Immunother..

[4] Chmielewski M, Abken H (2017). CAR T cells releasing IL-18 convert to T-Bet(high) FoxO1(low) effectors that exhibit augmented activity against advanced solid tumors. Cell Rep..

[5] Zimmermann K (2020). Design and characterization of an “all-in-one” lentiviral vector system combining constitutive anti-G_D2_ CAR expression and inducible cytokines. Cancers.

